# Opportunities and challenges of implementing Pharmacogenomics in cancer drug development

**DOI:** 10.20517/cdr.2018.22

**Published:** 2019-03-19

**Authors:** Paolo Tarantino, Dario Trapani, Stefania Morganti, Emanuela Ferraro, Giulia Viale, Paolo D’Amico, Bruno Achutti Duso, Giuseppe Curigliano

**Affiliations:** ^1^Division of Early Drug Development for Innovative Therapies, IEO, European Institute of Oncology IRCCS, Milan 20141, Italy.; ^2^Department of Oncology and Haematology (DIPO), University of Milan, Milan 20122, Italy.

**Keywords:** Pharmacogenomics, cancer drug development, precision medicine, clinical trials

## Abstract

Cancer drug development is a time and resources consuming process. Around 90% of drugs entering clinical trials fail due to lack of efficacy and/or safety issues, more often after conspicuous research and economic efforts. Part of the discarded drugs might be beneficial only in a subgroup of the study patients, and some adverse events might be prevented by identifying those patients more vulnerable to toxicities. The implementation of pharmacogenomic biomarkers allows the categorization of patients, to predict efficacy and toxicity and to optimize the drug development process. Around seventy FDA approved drugs currently present one or more genetic biomarker to keep in consideration, and with the progress of Precision Medicine tailoring therapies on individuals’ genomic landscape promises to become a new standard of cancer care. In the current article we review the role of pharmacogenomics in cancer drug development, underlying the advantages and challenges of their implementation.

## Introduction

Since the development and approval of the first targeted agents, oncology care has moved from a one-size-fits-all paradigm with pure histology-oriented approach toward a tailored treatment, selected upon molecular and clinical features, namely Precision Medicine (PM). Tumor staging and histology, classically considered the main factors influencing the therapeutic decision, where integrated by a large number of new identified prognostic and predictive biomarkers. Some of these where found to predict response or resistance to certain drugs (e.g., Estrogen receptor expression for Tamoxifen^[1]^, HER2 amplification for trastuzumab^[2]^), some others predicted instead a propensity in experimenting side effects from the treatments (e.g., UGT1A1 polymorphisms and irinotecan toxicity^[3]^). Both of these types of biomarkers are today studied by the discipline of *pharmacogenomics (PGx)*, which evolved from a pre-existing branch of pharmacology called *pharmacogenetics*. The term *pharmacogenetics* was originally coined by the German pharmacologist Friedrich Vogel, referring to polymorphisms of specific genes inducing individual drug response or susceptibility to adverse drug reactions. With the availability of genome-wide sequence data, and the advent of the “omics” era, this branch evolved into what we today call pharmacogenomics. As these terms are often used interchangeably in literature, in this text we’ll use the same abbreviation (PGx) as a referral to both.

Nowadays, several FDA-approved drugs contain pharmacogenomics information in their labeling, and more than seventy approved oncological drugs present one or more genetic biomarker to keep in consideration^[4]^. As we’re going to discuss further in the review, this circumstance is due to the fact that cancer PGx involves the study of both the germline genome and cancer’s somatically mutated genome. Both germline and somatic mutations can be, in different ways, responsible for the outcome of cancer treatments, and for this reason FDA’s Table of Pharmacogenomic Biomarkers includes both^[4]^.

## Pharmacogenomics in cancer drug development

### Pharmacogenomics in the medical fields

The discipline of PGx studies the relationship between specific DNA-sequence variation and the effect of drugs in terms of efficacy and toxicity. These variations include single nucleotide polymorphisms, deletions, insertions, tandem repeats, copy number variations and chromosomal translocations. Each of these changes can be germline or acquired: the first circumstance is common in drug-metabolizing enzymes, whose polymorphisms affect drug activation and excretion, or predictive biomarker of response, like BRCAs or mismatch-repair germline genes; the latter usually refers to accidental changes in DNA which ultimately cause cancer, with consideration of driver genomic alterations potentially *druggable*.

**Figure 1:**
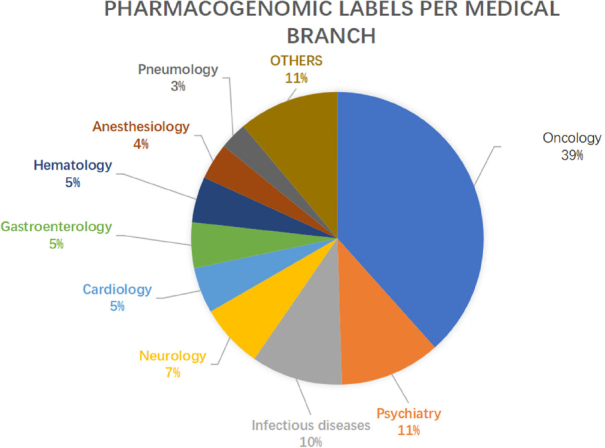
Distribution by field of FDA-approved drugs including pharmacogenomic information

PGx have led to an improvement in various fields of medicine, with special regard to Psychiatry, Neurology, Infectious diseases and Cardiology [Figure 1]^[5]^. One field benefiting greatly by the implementation of PGx biomarkers is Oncology, with almost 40% of the PGx labels concerning antineoplastic drugs. Many reasons lead to the success of this approach in Oncology: in this field, in fact, we more often find drugs with a narrow therapeutic index, which can lead to severe and sometimes life-threatening adverse events; identifying toxicity biomarkers is therefore critical. Moreover, the pipelines of developing oncological drugs have been extremely active lately, with a progressive increase of cancer drugs being evaluated in clinical trials^[6]^. Finally, while most of the medical disciplines are predominantly interested in germline alterations, the oncological field provides two different but related genomes to be studied, namely the somatic tumoral and germline genome, adding a factor of complexity and opportunity to PGx research. Indeed, the intensive study of cancer’s somatic mutations, together with improving our understanding of tumor development, provided a wide set of druggable alterations which ultimately explain why such a vast past of FDA’s Table is dedicated to Oncology. Nonetheless, each branch of medicine is moving toward a PM approach, and advancements in PGx are expected to affect the way all drugs are prescribed in a wider sense.

### Pharmacogenomics in cancer drug development

The application of PGx principles is clearly beneficial in the treatment of certain cancers^[7]^, but its role might be even more crucial in the setting of drug development. Cancer drug development is a time and resources consuming process: it takes around ten years for a new drug to complete its path from initial discovery to the approval, accounting for an average cost from research and development to market approval of approximately $2.9 billion^[8]^. Most of the resources are spent in the late phases of development, where large numbers are needed to confirm preliminary efficacy and toxicity data, and to confront them with the standard regimens. Despite huge investments, more than half of the drugs tested in phase 3 clinical trials fail to show a meaningful clinical benefit, due to three main reasons: inadequate antitumoral efficacy (57%) in term of survival gain, safety concerns (17%) with unacceptable toxicity profile and commercial reasons (22%) such as bankruptcy of the company^[9]^. Although large pharmaceutical companies loss investments can be at least partially absorbed, for smaller firms a single late-stage trial failure can be disastrous^[10]^.

**Figure 2:**
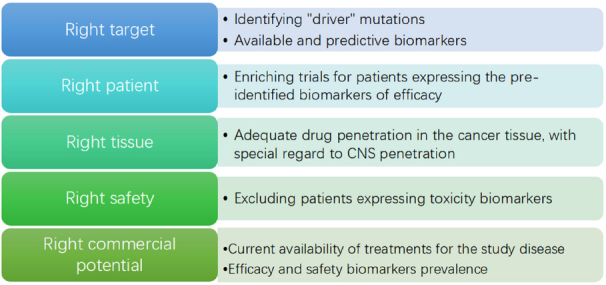
Proposal of a “five Rs” framework applied to cancer drug development

One crucial issue in cancer drug development is the discordance between Phase 2 and Phase 3 results: many promising drugs, for which Phase 2 results in terms of safety and toxicity seem solid, ultimately fail in Phase 3, sometimes even showing a detrimental effect. A recently published report by FDA^[11]^ underlined the relevance of this problem, observing that even large phase 2 studies assessing clinical outcome are too often disproved by Phase 3 studies. On the other hand, Pharma are actively studying the problem, in order to avoid the huge money and time losses which come with Phase 3 failures. A paper published in 2014 by AstraZeneca’s scientists analyzed nearly 150 drug development projects at the company, to identify cornerstones of trial failure^[12]^; “five Rs” where found to be the most important technical determinants of project success and pipeline quality: the right target, the right patient, the right tissue, the right safety and the right commercial potential. A recent update showed that the application of the “five Rs” improved success rates (from candidate drug nomination to phase 3 completion) from 4% in 2005-2010 to 19% in 2012-2016^[13]^ [Figure 2].

At least three of the 5 Rs (right safety, right target determination and right patient identification) can be improved by the implementation of PGx principles in cancer drug development, making it a main determinant of the success rate in clinical trials.

### The right safety: germline variants conditioning drug’s metabolization

Since its theorization in the late 1950s, the traditional role of PGx in medicine has been related to drug safety^[14]^: by identifying those genetic factors leading to an increased drug susceptibility, it was possible to better tailor pharmacological treatments. The most common identified factors where genetic variants influencing drug’s pharmacokinetics and pharmacodynamics: landmark examples are the discovery of thiopurine S-methyltransferase (TMPT) polymorphisms conditioning 6-mercaptopurine (6-MP) toxicity^[15]^ and uridine diphosphate glucuronosyltransferase 1A1 (UGT1A1) polymorphisms leading to irinotecan toxicity^[16]^. 6-MP is a commonly prescribed drug in the treatment of acute lymphocytic leukaemia; the individuation of germline genetic variants of TMPT inducing poor metabolization and increased toxicity lead to FDA recommendation of routinely genotyping this gene ahead of the therapy, to reduce 6-MP doses if an inactive allele is found^[16]^. Regarding irinotecan, certain polymorphisms of UGT1A1 are associated to increased toxicities in patients treated with this drug (irinotecan is commonly prescribed in the treatment of colorectal cancer and small-cell lung cancer), as a result of the increased concentrations of its active metabolite SN-38^[17]^. A similar case is the one of dihydropyrimidine dehydrogenase (DPD) deficiency, which leads to increased toxicity in case of treatment with 5-fluorouracil or capecitabine (two widely used drugs in the treatment of various cancers)^[18]^. A recently published trial showed that prospective DPD genotype-based dose reductions - in patients receiving fluoropyrimidine-based anticancer therapy - improved overall safety of the treatment, proposing this strategy as a new standard of care^[19]^. Such findings supported the idea of an earlier investigation of PGx signals, in order to avoid mistreatment of vulnerable patients and unnecessary withdrawal of promising agents or combinations. A recent example is the stratification for UGT1A1 genotype in a Phase 1 trial studying CAPIRINOX combination^[20]^, which found different toxicity rate and dose-limiting-toxicity according to UGT1A1 polymorphism, leading to different doses to employ during the Phase 2, and assuring the best safety for both strong and poor metabolizers, with no loss in terms of survival. In contrast with these attempts of biomarker-based personalization of doses, a different trend is arising on the drug market, with flat doses being promoted with the promise of shortening drugs preparation time and improving ease of administration (e.g., pembrolizumab was first approved for treating melanoma at the dose of 2mg/kg q3w, but a 200mg q3w dose was later approved and is now commonly prescribed). Although flat doses might not increase treatment toxicity, they can be associated to a substantial financial toxicity, since they promote the administration of an unneeded drug excess, which increases drug and treatment costs.

Despite their preeminent importance, germline genetic variants conditioning drug toxicities are found only in a minority of patients, with less then 10% of patients harboring deleterious TMPT polymorphisms^[17]^, approximately the same percentage of patients with deleterious UGT1A1 polymorphisms^[21]^ and less than 1% of patients being affected by DPD^[22]^. For this reason, such PGx labels are still scarcely implemented in clinical trials, with most of the studied biomarkers referring instead to somatic alterations in oncogenes and oncosuppressors. Therefore, for the purpose of this review, a major focus will be put on somatic PGx labels and the way they are changing the way we conduct clinical trials.

### The right target for the right patient: a genomic-oriented approach in Basket trials and Umbrella trials

One important application of PGx in cancer drug development regards molecularly targeted therapy, an approach deriving from the idea that targeting a driver molecular alteration found in cancer tissue might control cancer proliferation. This principle provided great improvements in cancer treatment, leading to a new paradigm of drug choice, molecular-alteration-centered rather than histology-oriented^[23]^.

New trial designs (the so called Master Protocols) have been developed in order to validate this approach; this term includes three different trial types: Umbrella trials, which study various targeted agents in the context of a single disease (e.g., NCI-MATCH trial); Basket trials, which study a single targeted therapy in the context of multiple diseases or disease subtypes (e.g., Braf V600 trial); Platform trials, which study multiple targeted therapies in the context of a single disease in a perpetual manner, allowing to add or remove therapies from the platform on the basis of a decision algorithm with an adaptive statistical design (e.g., I-SPY2)^[24]^. Such trials might accelerate the approval of targeted drugs, by hitting “the right target” in “the right patient” regardless of the histological type of tumor. By now, contradicting results have been obtained from molecularly driven trials, with the disappointing results retrieved from the Phase 2 SHIVA trial^[25]^ in contrast to the positive signals deriving from the NCI MATCH trial^[26,27]^ together with the success of the Phase 2 basket trial of larotrectinib in TRK-fusion positive patients^[28]^ and the demonstration of the activity of pembrolizumab in mismatch-repair deficient tumors^[29]^, which lead to their agnostic approval for any solid tumor type. Many answers are awaited from the results of the aforementioned Master Protocols, which might lead to the approval of more drugs with the same agnostic paradigm, avoiding the canonical phase 1 to phase 3 drug development, particularly in the genomic- oriented approach targeting rare genomic alterations, requiring huge efforts to be tested in randomized phase 3 histology- oriented trials.

### The role of Pharmacogenomics in early developmental phases

**Figure 3:**
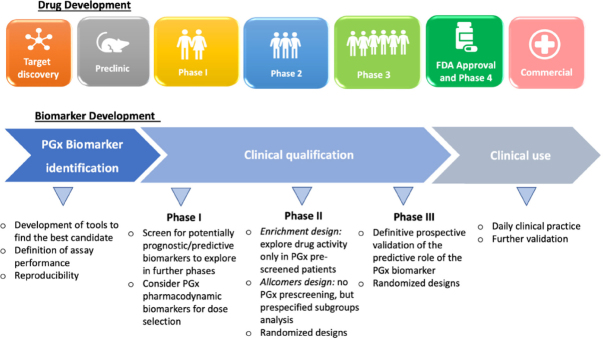
Possibilities of PGx implementation in each R&D phase

The adoption of PGx principles in cancer drug development can be beneficial in every phase of drug development, from compound discovery to post-marketing surveillance [Figure 3]. Although most of the PGx discoveries to date have emerged from phase 3 or post-marketing studies, evidence of a critical role of PGx in early-phase trials are arising^[30]^. The late implementation of PGx can in fact lead to discard potential useful drugs, or to the approval for unselected patients of drugs which are beneficial only in a subset of them: one clear example is the approval of cetuximab in 2004 for the treatment of unselected colorectal cancer patients, and the following discovery in 2006 of no-benefit, possibly detrimental, from the therapy in KRAS mutated patients^[31]^.

The traditional limitations of early-phase setting (small samples, short trial duration, variable doses) are being addressed by the emergence of new trial designs, which benefit of the implementation of PGx biomarkers in different manners. Not every early phase of Clinical Trials benefits in the same way of PGx implementation, and the role of PGx varies according to the target of the phase. For instance:

Traditional Phase 1 trials in the past aimed at exploring the safety of compounds, and finding the maximum tolerated dose for phase 2 development (recommended phase 2 dose, RP2D), by a progressive escalation of the administered doses trough what is known as the 3 + 3 design^[32]^. Until 2006, the vast majority of phase 1 trials would apply this classic design^[33]^. Nonetheless, it necessarily exposes a part of the patients to sub-therapeutic doses of the study drugs, thus posing ethical concerns. This consideration, together with the increase of targeted therapies and immunotherapies being investigated in clinical trials, provided the basis for the development of new early phase trial Designs. In the current era of targeted and immune therapies, the assumption that higher, more toxic doses of study drugs result in higher antitumor activity have been brought into question, leading to the new concept of “optimal biologic dose”. During the development of pembrolizumab, for example, despite toxicity studies reached doses up to 10 mg/kg without the observation of dose-limiting toxicities^[34]^, the selected dose for expansion trials (and finally for its approval) was 2mg/kg, based on translational models and pharmacokinetic/pharmacodynamics studies^[35]^. Such evidence promoted the adoption of new, Bayesian designs, allowing to more rapid dose escalation and the definition of a dose-toxicity curve to which all patients enrolled in the trial contribute. In the setting of dose-finding, the concept of *biomarker-based dosing algorithms* was proposed, based on the assumption that the PGx profile could help defining the optimal drug dose for each patient. Indeed, not every type of gene alteration is equally actionable, as it is exemplified by the different sensitivity of HER2-amplified and HER2-mutated cancers to anti-HER2 agents^[36]^; in this setting, Bayesian phase 1 and 2 study designs have been proposed to adapt drug doses to patient’s PGx profile^[37]^. The rise of more flexible study designs helped in the assessment of the role of biomarkers, even in the small cohorts of phase 1 studies: in the case of pembrolizumab, for instance, data regarding PD-L1 expression and activity in 15 patients informed later phases, leading to the cut-off identification of PD-L1 > 50% in NSCLC in a relatively short time. Generalizing this concept, even the small ratio of positive PGx findings of Phase 1 studies may be critical in contributing to more effective Phase 2-3 studies and shorter time to developmental decisions, orienting the investments and reducing the rates of patients less likely to derive a clinical benefit from drugs under development.

Phase 2 trials aim to assess drug efficacy, as well as further evaluate its safety. They enroll a higher number of patients, usually with a specific type of cancer and treated with fixed drug doses (the RP2D). All of these features make this setting the most promising for identifying efficacy biomarkers: O’Donnel and Stadler *et al*.^[30]^ report a 70% success ratio in identifying “positive” PGx findings (worthy of additional follow up and validation) in a cohort of 57 Phase 2 studies which incorporated PGx. PGx biomarkers can be implemented in different ways into phase 2 trials, depending on the characteristics of the biomarker. The *enrichment* design, for instance, screens patients for the presence or absence the PGx biomarker and then only includes patients who either have or do not have the marker in the clinical trial; this design is clearly beneficial when there is a strong preliminary evidence (e.g., deriving from phase 1 trial results) to suggest benefit only in the biomarker-defined subgroup, and/or when the marker prevalence is low (therefore the risk of diluting the drug effect by treating un unscreened population is high). In the *allcomers* design, instead, all patients meeting the eligibility criteria are entered the trial, regardless of the PGx marker status, but prospectively specified subgroup analysis of the treatment effect within biomarker-defined subgroups can be included to explore the predictive potential of the biomarker; this design is recommended when preliminary evidence on the biomarker is low, and when its prevalence in the population is high. Another key feature of phase 2 trials is the possibility to randomize patients. Since most phase 1 trials are single-arm trials with no control arm, prognostic and predictive values of PGx findings can’t be discerned in this setting; in the context of phase 2 trials, instead, there’s a growing trend in designing randomized phase 2 trials (in a 2008 review of targeted agents phase 2 trials, as many as 30% of the trials where randomized^[38]^). Randomization enhances the potential for biomarker discovery and the ability to discern the potential predictive value of previously discovered PGx findings, reducing the risk for bias existent in comparisons with historical controls^[39]^. PGx biomarker inclusion in the trial design, together with randomization of patients, represent powerful tools in the discovery and exploration of predictive PGx biomarkers, which can inform the design of confirmatory phase 3 trials in a more tailored and efficacious way.

### Challenges in Pharmacogenomics implementation

The implementation of PGx in cancer drug development, although undoubtedly promising, still needs to address a certain number of issues. First of all, obtaining enough tissue for genomic tests can be challenging. The identification of PGx biomarkers of safety and activity should ideally involve multiple biopsies, at drug initiation, during treatment and at disease progression. Although the first usually provides benefits to the patients, the two last are mainly meant to inform the researchers, exposing the patient to invasive techniques from which they derive no direct benefit, addressing a sensitive ethical question. This well-known issue has emerged more than ever in precision medicine trials, where the identification of mechanisms of resistance is crucial for the optimization of treatments^[40]^. In this setting, the promising alternative of liquid biopsy could provide the needed information with a simple and non-invasive blood draw. Landmark examples are the detection of T790M mutation in NSCLC treated with anti-EGFR^[41]^, the early detection of emergence of KRAS mutations in colorectal cancer^[42]^ and the detection of mutations reverting sensitivity to PARP-inhibitors in prostate cancer^[43]^. Liquid biopsy could solve another emerging issue related to precision medicine: it could catch the intrinsic intratumoral heterogeneity of cancer, providing an integrated picture of the mutational scenario of the neoplasia which could prioritize druggable alterations and guide therapeutic decisions^[44]^.

An important controversy is related to the meaning of mutational findings in tumors, most of which are expected to be passenger mutations, as opposed to fewer driver mutations promoting cancer growth and spread^[45]^. Frequency-based and function-based approaches are being applied to individuate driver mutations, and have recently lead to the publishing by ESMO of a scale of actionability of molecular targets (ESCAT scale)^[46]^ based on the strength of the evidence supporting them, which will serve as a base to interpret PGx findings and improve clinical trials, as well as clinical practice. The scale is meant in a plastic way, and enables mutations to be upgraded or downgraded in response to newly available data.

Time remains a concern: adding PGx profiling in the inclusion/exclusion criteria of trials harbors the risk of delaying the initiation of experimental drugs, leaving patients off-treatment for a longer time. Such issue is especially relevant when a centralized confirmation of the biomarker status is required: mutation detection or gene expression assays might require up to 10 business days from the shipping of the sample to the report results, and assays that require macro-dissection of FFPE slides may take even longer time. Although sequencing time is expected to fall with the evolution of NGS techniques, logistics remain the rate-limiting step of the equation, meaning that solving the issue will require innovative organizational improvements.

Finally, ethics remain an important issue; especially in the context of enriched trials: by treating only the population of biomarker-positive patients, no data is collected regarding the biomarker-negative ones; nonetheless, we know that many targeted drugs work regardless of a biomarker (e.g., anti-VEGF drugs^[47]^) and some biomarkers used for the treatment choice are far from being precise (e.g., PD-L1 for immunotherapy^[48]^). It’s therefore dangerous to exclude wide populations of biomarker-negative patients from the trials without solid data regarding biomarker predictivity. Such matter should be addressed before the initiation of clinical trials, and preclinical studies should strongly link the biomarker status to safety and activity, together with deeply elucidating the biology of the marker.

## Conclusion

The way we conduct clinical trials in oncology has been designed in an era where cytotoxic compounds were the only systemic agents available to treat cancer. The evolution of anticancer therapies, with the development of targeted drugs and more recently immunotherapies, has progressively changed the way we conduct clinical trials, leading to innovative trial designs, such as Basket and Umbrella trials. In this context, PGx offer a powerful tool to implement PM, thus enhancing safety and activity of study compounds by selecting the right drug for the right patient. Moreover, the implementation of PGx into enrichment study designs promises to accelerate drug development, leading to faster drug availability for patients and cost savings for the pharmaceutical companies. The trend toward enriching trials coincides with a progressive trend in genotyping patients in clinical practice^[49]^: diseases such as non-small cell lung cancer proved to benefit from wide NGS sequencing^[50]^, and with the recent advances of PM in other big killers (e.g., metastatic breast cancer^[51]^) the implementation of sequencing techniques in the clinical setting could expand. In this scenario, we expect that early PGx findings from clinical trials will become easier to translate into clinical practice benefits, and that, in the next future, medical oncologists will have the possibility to disclose the complex genomic landscape of patients before prescribing each anticancer drug.

## Declarations

### Copyright

© The Author(s) 2019.
